# Exome Sequencing Identifies a Mutation in EYA4 as a Novel Cause of Autosomal Dominant Non-Syndromic Hearing Loss

**DOI:** 10.1371/journal.pone.0126602

**Published:** 2015-05-11

**Authors:** Fei Liu, Jiongjiong Hu, Wenjun Xia, Lili Hao, Jing Ma, Duan Ma, Zhaoxin Ma

**Affiliations:** 1 Key Laboratory of Metabolism and Molecular Medicine, Ministry of Education, Department of Biochemistry and Molecular Biology, School of Basic Medical Sciences, Shanghai Medical College of Fudan University, Shanghai, China; 2 Department of Otorhinolaryngology, Shanghai East Hospital, Tongji University, Shanghai, China; 3 Institutes of Biomedical Sciences, School of Basic Medical Sciences, Shanghai Medical College of Fudan University, Shanghai, China; Universitat Pompeu Fabra, SPAIN

## Abstract

Autosomal dominant non-syndromic hearing loss is highly heterogeneous, and eyes absent 4 (EYA4) is a disease-causing gene. Most EYA4 mutations founded in the Eya-homologous region, however, no deafness causative missense mutation in variable region of EYA4 have previously been found. In this study, we identified a pathogenic missense mutation located in the variable region of the EYA4 gene for the first time in a four-generation Chinese family with 57 members. Whole-exome sequencing (WES) was performed on samples from one unaffected and two affected individuals to systematically search for deafness susceptibility genes, and the candidate mutations and the co-segregation of the phenotype were verified by polymerase chain reaction amplification and by Sanger sequencing in all of the family members. Then, we identified a novel EYA4 mutation in exon 8, c.511G>C; p.G171R, which segregated with postlingual and progressive autosomal dominant sensorineural hearing loss (SNHL). This report is the first to describe a missense mutation in the variable region domain of the EYA4 gene, which is not highly conserved in many species, indicating that the potential unconserved role of 171G>R in human EYA4 function is extremely important.

## Introduction

Hearing impairment is one of the most common losses of meaningful function in humans and poses a persistent threat to worldwide public health. Approximately 10% of people worldwide have mild or moderate hearing impairment [1]. Genetic factors are important for the pathogenesis of deafness, and non-syndromic hearing impairment (NSHI) accounts for approximately 80% of genetic deafness. Currently, 85 genes are known to play a role in NSHL (http://hereditaryhearingloss.org), and autosomal dominant inheritance accounts for approximately 20% of all NSHL cases.

Eyes absent (EYA) genes are members of a conserved network that regulates transcriptional and signal transductional activities and manipulates the development of the muscle, ear, eye and kidney [2–5]. Encoding a 639 amino acid protein, EYA4 is composed of a highly conserved 271 amino acid carboxy terminus called the eya-homologous region (eyaHR) and a more divergent proline-serine-threonine (PST)-rich transactivation domain at the amino terminus called the eya-variable region (eyaVR) [6]. Mutations in the EYA genes have been associated with several congenital diseases, including congenital cataracts [7], a multi-organ disease called bronchio-oto-renal syndrome [8] and late-onset deafness [9–13]. Previous studies have demonstrated that the EYA protein is a nuclear transcription factor that acts by interacting with homeodomain-containing sine oculis proteins [14]. The EYA protein is also a protein tyrosine phosphatase that does not resemble classical tyrosine phosphatases, which use cysteine as a nucleophile and proceed through a thiol-phosphate intermediate [15]. In contrast, the EYA protein is the prototype for a class of protein tyrosine phosphatases that use a nucleophilic aspartic acid in a metal-dependent reaction [16]. The eyaHR and sine oculis homeobox (SIX) family of transcription factors interact to form transcriptional complexes that regulate the expression of target genes that are required for the development and maturation of the organ of Corti [9].

Mutations in the EYA4 gene cause inherited DFNA10 autosomal dominant hearing loss [9] and share a similar phenotype: postlingual onset, with the age of the first attack varying from 6 to 50 years old; progressive, sensorioneural hearing loss (SNHL) at first, with hearing loss in the middle frequencies and tinnitus as the most common complaints; and continued SNHL, with all frequencies become involved as age increases and the degree of hearing loss gradually evolving from mild to moderate and severe [17]. Thus far, 7 mutations of the EYA4 gene with SNHL have been identified, and most of the reported mutations affect the eyaHR of the EYA4; no missense mutation has been found that affects the eyaVR [18].

In this study, we used whole-exome sequencing (WES) in a family with SNHL and ultimately identified a novel missense mutation in the eyaVR of the EYA4, which is not highly conserved among many species, indicating that the potential role of the variable region of the EYA4 function is also extremely important.

## Materials and Methods

### Family Recruitment and Clinical Evaluations

**Fig 1:**
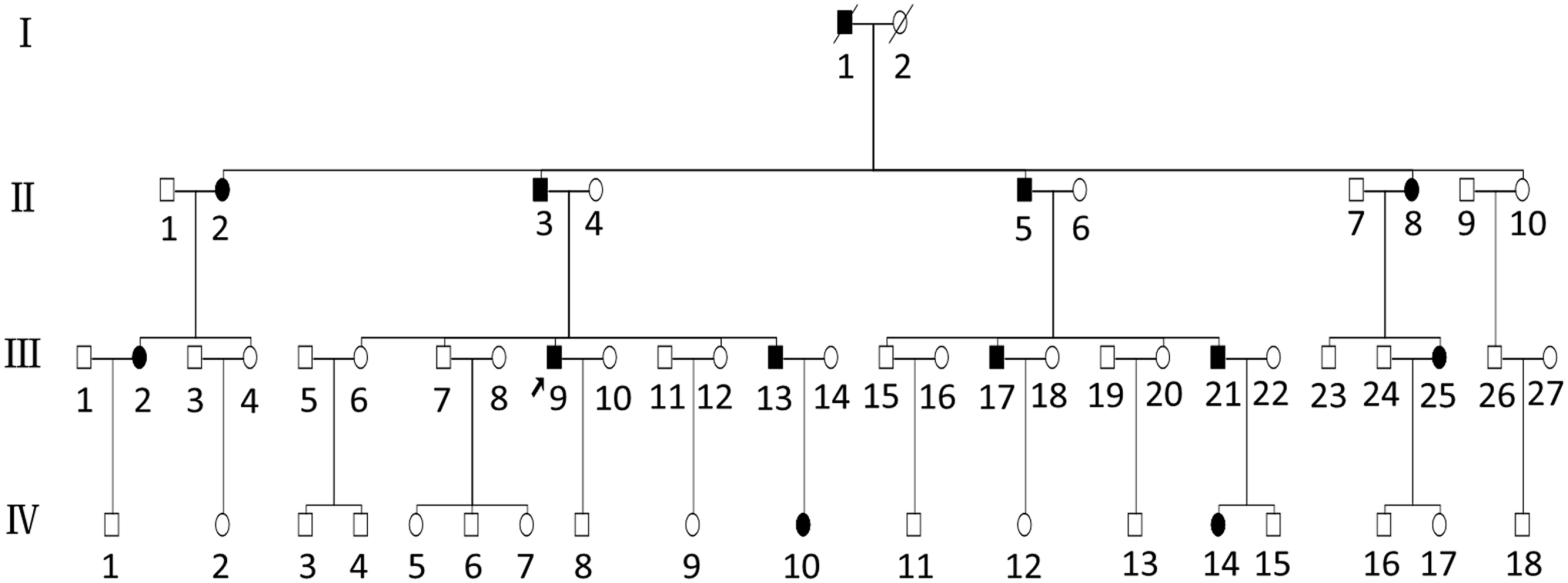
Partial pedigree of the Chinese family with nonsyndromic autosomal dominant SNHL. Open symbols denote unaffected individuals; filled black symbols denote affected individuals. The arrow labels the proband.

A four-generation family (SH-01) with 57 members presenting with segregating AD-NSHL was identified by the Department of Otolaryngology, Head and Neck Surgery of Shanghai East Hospital, Tongji University, Shanghai, China (Fig 1). All clinical information was collected at the Department of Otolaryngology and Head and Neck Surgery, Shanghai East Hospital, Tongji University, Shanghai, China. Medical histories were obtained using a questionnaire covering the following issues: subjective degree of hearing loss, age at onset, evolution, symmetry of hearing impairment, use of hearing aids, presence of tinnitus and vertigo, medication, noise and ototoxic drug exposure, pathological changes in the ear, and other relevant clinical manifestations. Systemic medical examinations and approximate intelligence assessments were also performed on all affected individuals. Another 237 sporadic cases of hearing loss participated in this study, along with 500 ethnically matched controls. All procedures were approved by the Ethics Committee of Shanghai East Hospital, which is associated with Tongji University, and were carried out only after written informed consent had been obtained from all study participants and from the parents of subjects younger than 18 years, who were informed that all data collected will only be used for scientific research and not for any commercial purpose.

### Audiological tests and imaging studies

Audiological tests were performed in a standard anechoic chamber with a pure-tone audiometer (Interacoustics, AD229b, Manufactured by Interacoustics A/S DK-5610 Assens Denmark) at frequencies ranging from 250–8000 Hz. Using an acoustic emmittance measurement apparatus (Interacoustics, AT235 h, Manufactured by Interacoustics A/S DK-5610 Assens Denmark), ABR was recorded ipsilaterally in response to click stimuli presented at 90 dBnHL (ABR, Interacoustics, Eclipse EP2, Manufactured by Interacoustics A/S DK-5610 Assens Denmark), and DPOAE (2f1–f2) (Interacoustics, DPOAE20+TEb, Manufactured by Interacoustics A/S DK-5610 Assens Denmark, f2/f1 = 1.22; the level for f1 was 65 dB spl and the level for f2 was 50 dB spl. DP S/N: 5 dB SPL) was used. Ear endoscopy, CT scans, and MRI were used to exclude deafness caused by anatomical abnormalities of the middle and inner ear.

### Whole-exome sequencing

Genomic DNA was extracted from whole blood samples using a blood DNA kit (Qiagen, Germany), and 1 μg of purified gDNA was fragmented to 200 base pairs. End repair, adenylation and adapter ligation were performed for library preparation following the Illumina protocol. Equal amounts of library samples were pooled and then hybridized to the customized capture array, including exons, splicing sites and immediate flanking intron sequences. Sequencing was performed on an Illumina HiSeq 2500 to generate paired end reads.

### Sanger sequencing

Sanger sequencing was used on samples from all available members from the SH-01 family to determine whether the potential mutations in causative genes co-segregated with the disease phenotype in these families. The PCR products were sequenced using BigDye Terminator v3.1 Cycle Sequencing Kits (Applied Biosystems, Foster City, CA, USA) and analyzed using an ABI 3700XL Genetic Analyzer.

### In silico analysis

In the present study, we used SIFT, Polyphen2 and MutationTaster software to determine possible changes in the protein structure that might affect the phenotype. Clustal X1.83 software was used to compare the human wild-type EYA4 protein sequence with the orthologs from *Homo sapiens*, *Pan troglodytes*, *Macaca mulatta*, *Mus musculus*, *Mus musculus*, *Gallus gallus*, *Takifugu rubripes*, *Danio rerio* and *Xenopus tropicalis* and to examine evolutionary conservation and structural prediction for this protein (sequences obtained from http://www.ensembl.org/).

## Results

### Clinical description

**Fig 2:**
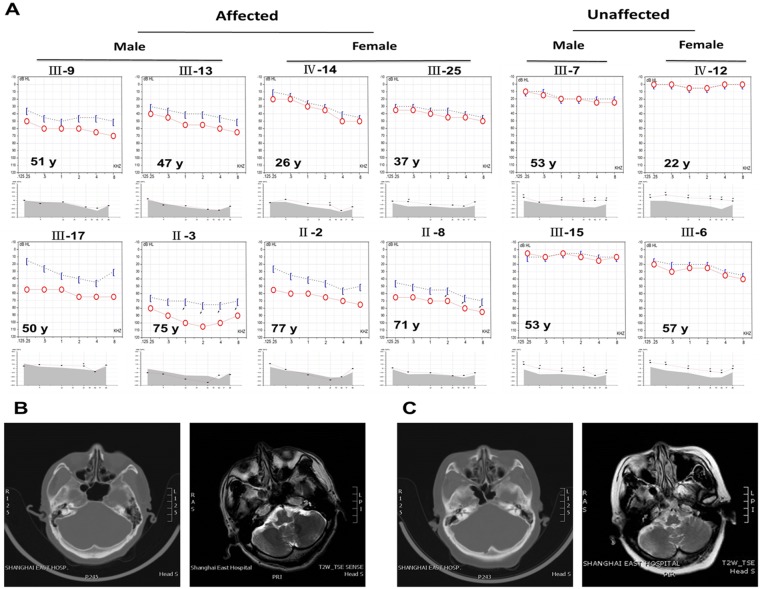
(A) SH-01 audiometric phenotype. Pure-tone bone and air conduction thresholds as well as otoacoustic emission are presented for the right ears of the SH-01 family members. Two representative stages (early and late) are presented, with corresponding audiograms. Blue half-frame and open, red circles indicate the bone and air conduction thresholds, respectively, for the indicated ages, from youngest to oldest. Both conduction thresholds were consistent with predominant SNHL, and some individuals at the later stage displayed conductive hearing loss. In the family, Otoacoustic emission could be induced at most frequencies in individuals with normal hearing, while it was the reverse in affected individuals. (B, C) Based on the CT scan and MRI data of the proband (III9) and his aunt (II2), the mastoid process and cochlea were well developed, and the ossicular chain was intact. Additionally, both the internal auditory meatus and the membranous labyrinth were well developed.

For the proband, the initial onset of hearing loss occurred at age 30 along with tinnitus but not vertigo. Pure tone audiometry displayed a prominent sensorineural and slight conductive hearing impairment at the middle and high frequencies (0.5–8 kHz, particularly 1–4 kHz), and the auditory threshold of air conduction measurements was 50–60 dBHL, yielding an audiogram with a “gentle slope” configuration (Fig 2A). Tympanometry indicated a type C curve of tympanogram, demonstrating an impaired eustachian tube function (the threshold of acoustic reflex was 100 dBHL.) The Metz recruitment test appeared negative at the low frequencies and positive at the middle and high frequencies. Otoacoustic emission could not be induced at any frequency. In the AS for the auditory brainstem response (ABR) with a click stimulus of 100 dBHL, both ears displayed well-differentiated wave profiles and regular latency, which indicated that no retrocochlear disorders were present. Of the 57 individuals in this 4-generation family (2 of whom had passed away), 13 complained of hearing loss (1 of whom had passed away). These individuals ranged in age from 26 (IV: 14) to 77 (II: 2) and were evenly distributed in the four generations, and each displayed approximately the same age of onset (ages 26–32 years). All of the patients had normal auditory senses and verbal function before the onset of the disease. No visible gender difference in the incidence of this disease was observed. During the early stages of disease, the primary symptoms were tinnitus and middle and high frequency hearing loss, which progressively deteriorated into whole-frequency hearing loss, presenting a “gentle slope” audiogram during all stages of the disease (Fig 2A). Some patients in the later stage also displayed conductive hearing impairment (air-bone gap>10 dB). Otoacoustic emission could not be induced at most frequencies (Fig 2A). Upon ABR examination, both ears showed well-differentiated wave profiles and regular latency, which indicated that there were no retrocochlear disorders (Table 1). The clinical and instrumental evaluations did not reveal any evidence of syndromic features, such as cardiovascular diseases, diabetes, visual problems, or neurological disorders. All patients displayed normal intelligence. None of the patients had any history of constant exposure to noise or to ototoxic drugs (Table 2). Both the auricle and external auditory meatus developed normally, and the tympanic membranes were intact. Six patients presented with the type A curve of a normal tympanogram; 1 patient (II-8) presented with the type Ad curve of tympanogram; 5 patients (II-2, II-3, III-9, III-13, III-17) in the later stage of this disease displayed the type C curve of tympanogram. According to the CT scan and MRI data (Fig 2B and 2C), the mastoid process, cochlea, internal auditory meatus, and membranous labyrinth were also well developed, as was the ossicular chain.

**Table 1:** The ABR absolute latencies of wave I, III and V and the interpeak latencies (IPLs) I–III, III–V and I–V in the right ears.

Patient	Absolute latency(ms)					
	I	III	V	I-III	III-V	I-V
II-2	1.70	3.07	4.93	1.37	1.87	3.23
II-3	1.77	2.80	4.40	1.03	1.60	2.63
II-5	1.53	3.63	5.50	2.10	1.87	3.97
II-8	1.53	2.67	4.53	1.13	1.87	3.00
III-2	1.47	3.93	5.50	2.47	1.57	4.03
III-9	1.77	3.73	5.33	1.97	1.60	3.57
III-13	1.60	3.87	5.37	2.27	1.50	3.77
III-17	1.73	3.70	5.43	1.97	1.73	3.70
III-21	1.17	3.87	5.67	2.70	1.80	4.50
III-25	1.33	3.70	5.50	2.37	1.80	4.17
IV-10	1.37	3.53	5.40	2.17	1.87	4.03
IV-14	1.60	3.90	5.37	2.30	1.47	3.77

The wave profiles were well differentiated, and all of the latencies were regular.

**Table 2:** Summary of the audiological features of affected members of family SH-01.

Patient	Gender	Test age(years)	Onset age(years)	Use of aminoglycoside	PTA of left ear(dBHL)	PTA of right ear(dBHL)	Audiogram shape	Level of hearing loss	Vertigo	Tinnitus
II-2	Female	79	28	No	63.75	65	Gently sloping	Severe	No	Yes
II-3	Male	76	30	No	98.75	93.5	Flat	Severe	No	Yes
II-5	Male	74	30	No	75	75	Gently sloping	Severe	No	Yes
II-8	Female	71	31	No	71.25	75	Gently sloping	Severe	No	Yes
III-2	Female	59	29	No	65	65	Gently sloping	Severe	No	Yes
III-9	Male	51	31	No	61.25	62.5	Gently sloping	Moderate	No	Yes
III-13	Male	48	29	No	53.75	55	Gently sloping	Moderate	No	Yes
III-17	Male	52	33	No	60	55	Gently sloping	Moderate	No	Yes
III-21	Male	50	28	No	45	50	Gently sloping	Moderate	No	Yes
III-25	Female	49	31	No	41.25	42.5	Gently sloping	Moderate	No	Yes
IV-10	Female	26	26	No	45	40	Gently sloping	Moderate	No	Yes
IV-14	Female	29	28	No	33.75	40.25	Gently sloping	Moderate	No	Yes

The degree of hearing loss was defined according to pure-tone averages (PTA), which were based on the following four frequencies: 0.5, 1, 2 and 4 kHz.

### Whole-exome sequencing

**Fig 3:**
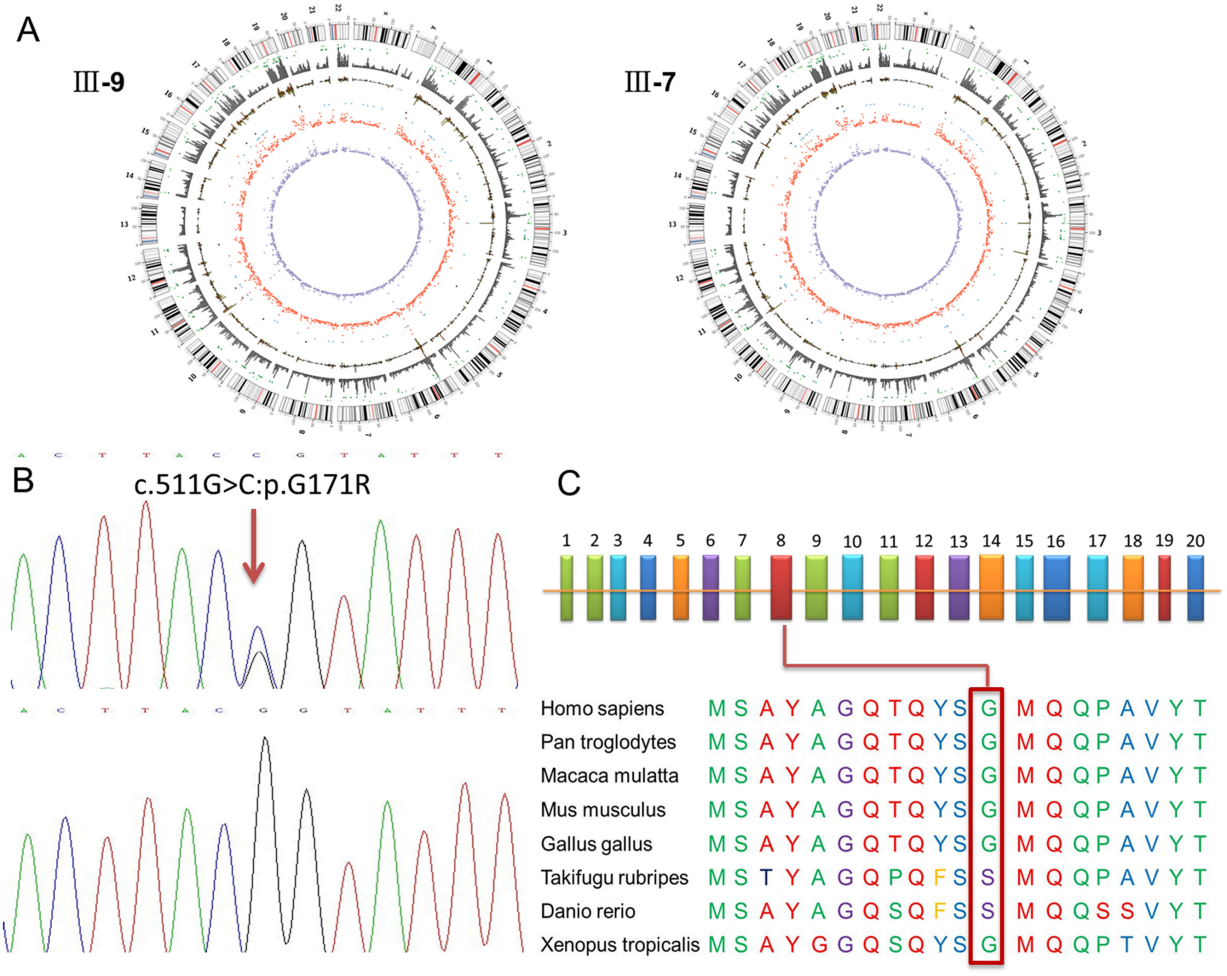
(A) Read coverage of each chromosome and mutation loci statistical results of the patients and of the normal control; (B) DNA sequence chromatograms presenting the two heterozygous missense mutations c.511G>C; p.G171R in affected individuals (upper panel) compared with the wild-type controls (lower panel). (C) The structure of the EYA4 gene. The EYA4 gene has 20 exons. The c.511G>C; p.G171R mutation identified in the EYA4 gene is in exon 8. The conservation analysis indicates that the Gly residue at 171 in the EYA4 protein is not conserved across *Homo sapiens*, *Pan troglodytes*, *Macaca mulatta*, *Mus musculus*, *Gallus gallus*, *Takifugu rubripes*, *Danio rerio* and *Xenopus tropicalis*.

To systematically search for deafness susceptibility genes, we performed WES on samples from one unaffected (III-7) and two affected (III-9 and III-13) individuals from the SH-01 family pedigree (Fig 1). An average of 4.93 billion bases of high-quality sequence was generated per individual, with an average sequencing depth of approximately 98 in the target region, which satisfied the requirements for calling single nucleotide polymorphisms (SNPs) and indels. The read coverage of each chromosome and the statistical results for mutation loci are shown in Fig 3A. The outer rings represent the chromosomes, arranged in a clockwise direction, and the location of the centromere is denoted with a red line. The outer gray rectangles indicate the read depth along the chromosome. The dark green points between the two circles denote deletion sites, and the light green points denote insertion sites. The rest of the mutation types, from the outside to the inside, are as follows: homozygous SNPs (orange rectangle), heterozygous SNPs (yellow rectangle), terminator codon mutation (black dot), nonsense mutation (blue dot), synonymous mutation (red) and missense mutation (purple). The sequencing data were aligned to the NCBI human reference genome (NCBI build 36.3, hg18) and compared with dbSNP138, which contains pilot data from the 1000 Genomes Project, from eight sequenced HapMap individuals, and 10 from the YH database, which contains the first whole-genome sequence of an Asian, individual.

In total, 9223 variants were identified in the two patients; 5789 of these were nonsynonymous variants, splice acceptor and donor site mutations and coding indels that were more likely to be pathogenic mutations per subject. Next, these variants were prioritized for further evaluation using two filtering criteria: if a variant was within the allele frequency cutoff, which was less than 0.01 in the dbSNP138, HapMap, 1000 human genome, and the local dataset, and if a variant was found in all of the affected individuals but not in unaffected family members, then this variant was retained for further analysis. These filtering criteria reduced the list of candidate variants to 16 non-synonymous mutations.

Then, we screened 16 variations found among the pedigree samples by Sanger sequencing and found a missense variant, c.511G>C; p.G171R, in exon 8 of EYA4 [NM_172105.3, (MIM*603550)], which co-segregated with the disease. This novel mutation was exclusively identified in all 12 affected patients but was not found in the 20 unaffected family members. To assess the possibility that this novel EYA4 mutation is a disease-causing mutation, we further sequenced 500 ethnically unrelated healthy individuals and confirmed that none of these 500 healthy donors carried the G171R mutation. Thus, our data suggested that this novel G171R missense mutation in the EYA4 gene was a disease-causing mutation in the Chinese pedigree (SH-01) with NSHL.

### In silico analysis

**Fig 4:**
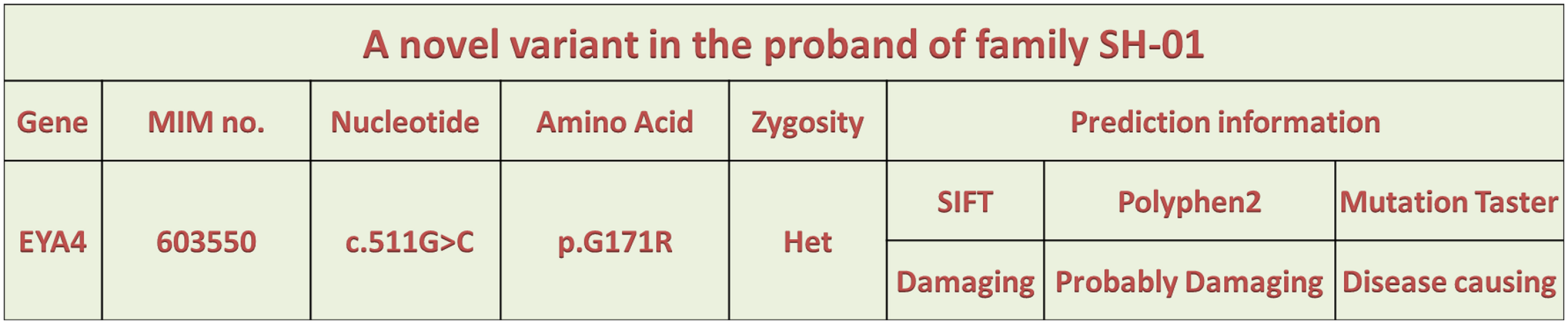
A novel variant was tested for pathogenicity using the bioinformatics software SIFT, PolyPhen2 and MutationTaster.

To understand the potential effect of the 171G>R missense mutation on EYA4 function, we further performed *in silico* analyses. This mutation was predicted to be “Damaging”, “Probably Damaging” and “Disease-causing” by SIFT, Polyphen2 and MutationTaster as shown in Fig 4, respectively. This finding indicated that this novel mutation might be the cause of the observed hearing loss in this Chinese family.

## Discussion

In 2013, the World Health Organization estimated that 360 million people worldwide live with disabling hearing loss and that as the population ages, the global burden of disease attributable to deafness increases [19]. Traditional methods of screening new disease-causing genes are expensive and time consuming; recently, sequencing technology has remarkably progressed. WES offers a convenient and fast method for finding new genes. In this study, WES was used to find the disease-causing gene of a large Chinese family with hearing loss, and we identified the EYA4 exon 8 missense mutation in all cases.

EYA4, which is an EYA family member, encodes a member of the EYA family of proteins. The encoded protein may act as a transcriptional activator through its protein phosphatase activity and may be important for eye development and for the continued functioning of the mature organ of Corti. Mutations in this gene are associated with postlingual, progressive, autosomal dominant hearing loss at the deafness autosomal dominant non-syndromic sensorineural 10 locus. This encoded protein is also a putative oncogene that mediates DNA repair, apoptosis, and innate immunity following DNA damage, cellular damage, and viral attack. Defects in this gene are also associated with dilated cardiomyopathy. EYA4 knockout mice suffer from serious hearing loss and secretory otitis media, and malformations of the tympanic cavity and auditory tube were found in anatomical studies [20].

**Fig 5:**
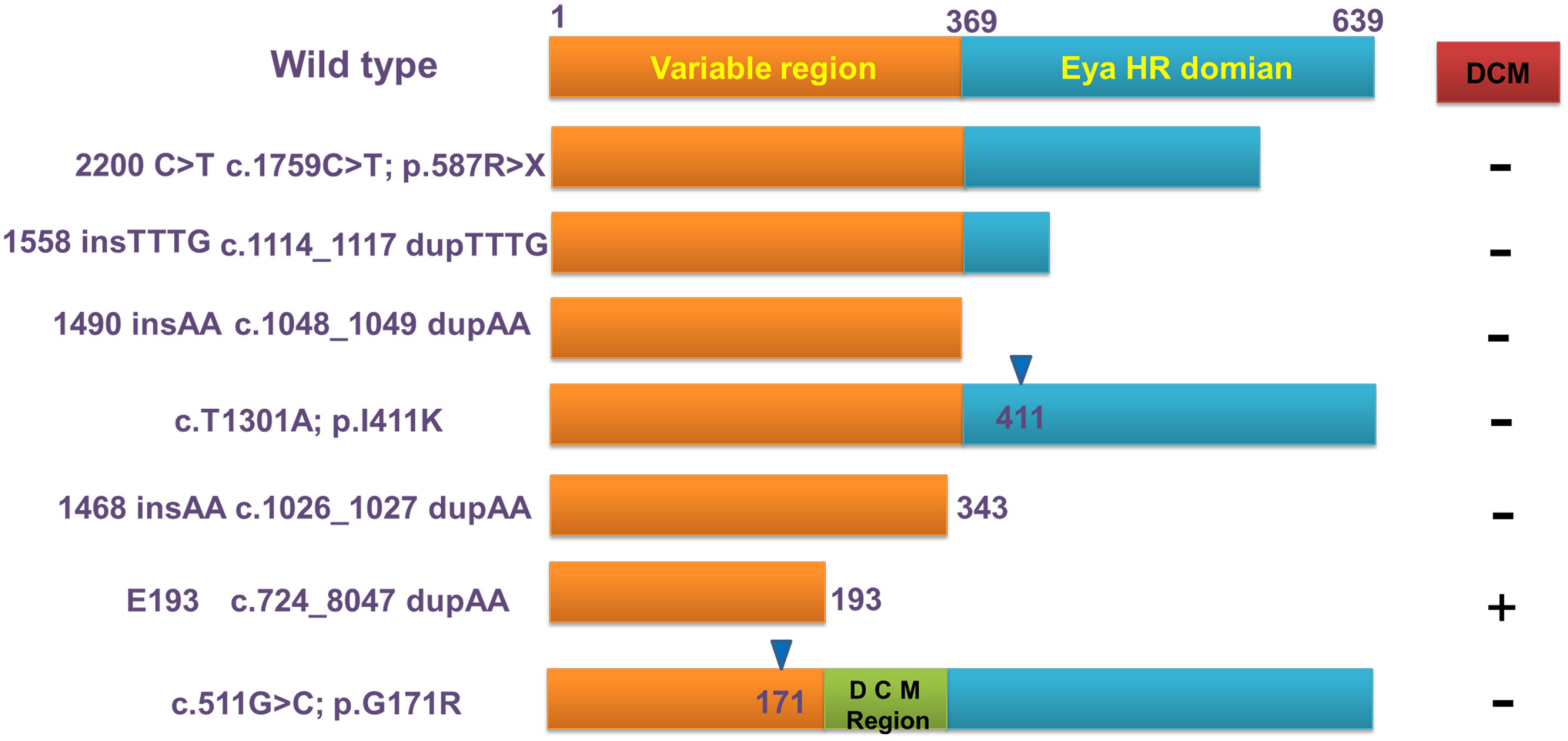
The effects of known EYA4 mutations on the EYA4 protein structure and on the cardiac phenotype. The number of amino acids of each allele product is indicated. Mutations that truncate the C-terminal Eya domain are associated with DFNA10 hearing loss and with a normal cardiac phenotype, whereas E193 truncates the N-terminal variable region and results in hearing loss plus dilated cardiomyopathy. Asp171 is in the variable region domain, and patients with this mutation presented with hearing loss and a normal cardiac phenotype.

The EYA4 protein is composed of 639 amino acids with 2 critical domains, including a highly conserved 271 amino acid C-terminus called eyaHR (alternatively called the eya domain or eya homology domain 1) and a more divergent proline-serine-threonine (PST)-rich transactivation domain at the N-terminus. To date, only 7 mutations of the EYA4 gene have been identified as shown in Fig 5. In 2 cases, the EYA4 mutations were indels, leading to frameshifts, and the other 2 cases were nonsense mutations [8, 9]. In addition, a family with dilated cardiomyopathy and SNHL was found to have a large deletion in EYA4 that also led to a frameshift [8]. Furthermore, a novel splice site mutation was identified in a five-generation Australian family. This report is the first describing a point mutation in the EYA4 gene that leads to aberrant pre-mRNA splicing. Similar to the four previously characterized EYA4 mutations, this variation is predicted to affect the eyaHR domain [11]. Recently, a missense mutation in the eyeHR domain was found in a Chinese family. Many pathogenic mechanisms have been presumed to be involved in NSHL caused by EYA4 mutations, including failure to interact with members of SIX and DACH protein families through a conserved network that regulates the early embryonic development and post-natal physiological function of the organ of Corti [9]. Defective apoptotic activity caused by EYA4 mutations may also lead to human DFNA10 hearing loss [10]. It was reported Eya4-deficient mice developed heritable otitis media, which indicated that Eya4 regulation is critical for the development and function of the middle ear cavity and eustachian tube[20]. This may explain the result that some of the patients in this family, particularly in the later stage, presented a type C curve of a normal tympanogram, demonstrating the abnormal physiological function of the eustachian tube. Through Na^+^/K^+^-ATPase regulation[21], EYA4 may participate in the development and maintenance of the hair cells of the inner ear, and analogously, the maintenance of ciliary cells distributing in the inner wall of the eustachian tube could also be impaired by EYA4 mutations, leading to defected tube function and conductive hearing loss[22].

The phenotype of EYA4-causing syndromic or non-syndromic deafness is correlated with the position of the mutation. Variations affecting eyaHR cause SNHL alone, whereas E193 truncates both eyaVR and eyaHR, resulting in DFNA10 plus DCM [12, 23]. Sigrid Wayne et al. found that a 1468insAA mutation in an American family causes a frameshift and a subsequent novel stop codon in amino acid 343; this mutation was found in the variable region without causing DCM [9]. Asp171, the residue affected in our study, is in the variable region domain, and patients who presented with mutations of the affected residue suffered from hearing loss and had a normal cardiac phenotype. Thus, the concept that the eyaVR mutation can cause DCM is somewhat questionable. Our result indicates that the type of mutation and the affected position within the scope of the variable region will lead to different phenotypes. In addition to the nonsense mutation of E193, this family presented with a missense mutation. Because the E193 mutation truncated amino acids 193–639, we can speculate that the region from 193–343 might play a key role in the maintenance of cardiac function. Because Asp171 is in upstream of site 193, the finding that Asp171 merely leads to hearing loss is not surprising.

The EyaVR domain regulates the eyaHR domain and combines with members of the SIX and DACH protein families in a conserved network that regulates early embryonic development and, post-developmentally, regulates the continued function of the mature organ of Corti [6, 9, 24].

There is a great deal of evidence that Six1 plays an important role in the development of the inner ear through its function as a transcriptional repressor or activator [23, 25]. It lacks an activation domain and requires interaction with EYA family members for transcription activation. The encoded protein, EYA4, could act as a transcriptional activator for SIX1 through its protein phosphatase activity. The mutation that we found in EYA4 may impair the formation of the complex that is regulated by EyaVR, which impedes complex transportation to the nucleus, where is acts as a transcription factor, and the haploinsufficiency of EyaVR may lead to inadequate cochlear transcriptional regulation and function maintenance and result in SNHL.
